# Twinning International Pediatric Cardiology Fellowship Programs: A Transformative Educational Experience for Trainees with Potential for Global Adoption

**DOI:** 10.1007/s00246-024-03469-x

**Published:** 2024-04-02

**Authors:** Sean T. Kelleher, William B. Kyle, Daniel J. Penny, Jillian Olsen, Lars Nolke, Hugh D. Allen, Colin J. McMahon

**Affiliations:** 1 Department of Paediatric Cardiology, Children’s Health Ireland at Crumlin, Dublin, 12 Ireland; 2 Department Pediatric Cardiology, Texas Children’s Hospital, 6621 Fannin Houston, Texas, 77030 USA; 3 Department of Congenital Cardiothoracic Surgery, Children’s Health Ireland, Crumlin, Dublin, 12 Ireland; 4 UCD School of Medicine, Belfield, Dublin, 4 Ireland; 5 Maastricht School of Health Professions Education, Maastricht, Netherlands

**Keywords:** Congenital heart disease, Education, Training, Pediatric cardiology fellowship program, Twinning, Collaboration

## Abstract

Over the last decade, having endured the COVID-19 pandemic, education and training in pediatric cardiology have undergone a profound disruptive transformation. Trainees experience considerable stress achieving all the competencies required to become a competent pediatric cardiologist. Often the quality of the training experienced by trainees, the approach to patients, and potential institutional preference in management strategy is heavily influenced by the center in which they train. We developed an online live twin program of education between Texas Children’s Hospital, Houston, Texas and Children’s Health at Crumlin Dublin Ireland in 2019. We explored using grounded theory whether a regular scheduled shared teaching program improved fellow education and training between both centers. Trainees were surveyed to evaluate the benefits and disadvantages of such a twin program. The majority (93%) found the sessions helpful from an educational standpoint with many trainees reporting it to be a transformative experience. Three important learning themes emerged: practice variation between centers, managing uncertainty in clinical practice and cognitive overload. This pedagogical model could be replicated across multiple international pediatric cardiology units and facilitate “collaborative learning” among centers across the globe. Furthermore, this novel educational model could also be adopted by other medical specialties.

**Supplementary Information:**

The online version contains supplementary material available at 10.1007/s00246-024-03469-x.

## Introduction

**Fig. 1:**
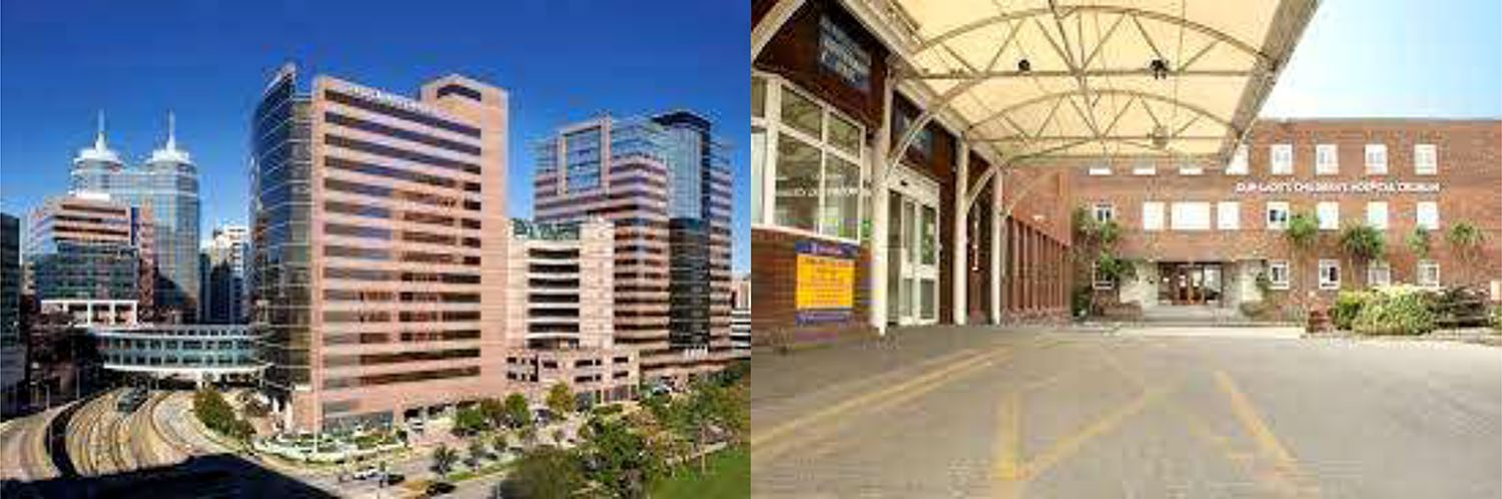
Twinning partnership between international pediatric cardiology training programs. Texas Children’s Hospital, Houston, Texas, USA (left panel) and Children’s Health Ireland at Crumlin, Dublin, Ireland (right panel)

Since 2019, an educational partnership has developed between pediatric cardiology fellowship programs at Texas Children’s Hospital (Houston, Texas, USA) and Children’s Health Ireland (Dublin, Ireland) (Fig. 1). Since the COVID-19 pandemic, there has been an unprecedented willingness to embrace online learning formats, such as webinars, with widespread success. While not without challenges, webinars may enhance attendance and participation as well as promote deep learning [1]. This partnership centers around co-chaired online educational sessions conducted on an alternate monthly basis in a webinar format. Each session has a didactic lecture or case presentation portion presented by a fellow, followed by a discussion chaired by experienced facilitators and, finally, a question-and-answer session.

### Building a Partnership

The concept of “twinning” is a collaborative model of partnership between two communities in which resources, knowledge, and staffing are shared in order to achieve a common goal. Such partnerships were originally developed by the European Union to strengthen relationships between member states [2]. It has been successfully utilized within medical education, including, admirably, integrated curricular development in low-income countries [3]. This partnership has been developed over 4 years. The two institutions are distinct in several aspects (Table 1). The pediatric cardiology program at Texas Children’s Hospital is considerably larger in terms of both permanent staff and patient numbers. Contextually, per population, Ireland has one of the lowest numbers of pediatric cardiologists in Europe (1.8 per million population) [4] and significantly less than in the USA (8.8 per million population) [5]. With a view to training and retention of trainees within Ireland, the Higher Specialist Training Scheme in Pediatric Cardiology under the auspices of the Royal College of Physicians of Ireland was established in 2014 [6]. It has since expanded its remit working with the All-Island Congenital Heart Network Program to provide training in both The Republic of Ireland and in Northern Ireland. The training program in Ireland is in its relative infancy compared with the larger program at Texas Children’s Hospital which was established in 1954 with the Board Examination in Pediatric Cardiology facilitated by the American Board of Pediatrics established in 1961 [7]. A comparison of assessment and accreditation is included in Table 1.

**Table 1:** Comparison of structure of twin centers

	Dublin	Houston
Staff		
Surgeons	3	8
Cardiologists (including interventionalists)	9 (CHI at Crumlin) 5 (Royal belfast hospital for sick children)	68
Interventionalists	3	7
Fellows per year	1–2 *Additional pediatrician with expertise fellow 1*	7 *Additional 7 sub-specialty fellows*
Total categorical Fellows	6	20
Surgeries (annually)	448	1150
Cardiac catheterization cases (annually)	654	1450
Program		
Year department founded	1954	1954
Year training program	2014	1954
Size of population served	5.28 million Republic of Ireland [8]	Houston 2.2 million
	1.90 million Northern Ireland [9]	Harris County 4.7 million
Public/private	Publicly funded	Privately funded
	Limited private out-patient service	
Fellowship		
Methods of assessment	- ePortfolio/logbook	- Evaluation after rotation block (13 blocks per year)
	- 3-monthly review with supervisor	- Twice yearly review of evaluations
	- End of year review with RCPI	- Evaluations from patient families
	- Twice yearly OSCE assessment	- Conference attendance
	- Programmatic assessment	- In-training exams
		- Research productivity
Accreditation	- Certificate of Successful Completion of Specialist Training (CSCST) awarded following successful completion of yearly formative reviews and final summative review	- Demonstration of competence in the Accreditation Council for Graduate Medical Education in the USA (ACGME) established aspects of pediatric cardiology
	- *Recommended: European Association of cardiovascular imaging certification in congenital heart disease echocardiography*	- Completion of a scholarly work
	- *Recommended: AEPC exam in Pediatric and Congenital Cardiology*	- Board Examination in pediatric cardiology under the American Board of Pediatrics

Despite their structural differences, the two institutions share several visions and goals. The first and foremost is to provide excellent care to infants and children with congenital and acquired heart disease. Through personal and professional links, the educational partnership was conceived by Dr. Colin McMahon (CHI), Prof. Daniel J. Penny (TCH), and Dr. Hugh D. Allen (TCH) with a view to building bridges of collaboration between international centers.

### Strategy Development

The educational strategy was developed from an understanding of the current challenges facing both trainees and educators within pediatric cardiology. Educators seek to produce trainees that are grounded in general pediatric cardiology so that they may safely treat common conditions [4]. The ideal acquired competencies of a trainee at completion stage vary in their specifics between countries. While there are calls for more internationally standardized approaches [10], desired competencies center around broad medical knowledge, technical mastery (particularly in echocardiography), and an increasing emphasis on exemplary communication skills in challenging scenarios [6, 11–13]. The content of the sessions is directed, not only, to cover central topics but also to encourage participants to think critically in a highly complex specialty in which large clinical trials are lacking. This is facilitated through discussions of cases that highlight decision-making and contrasting management strategies between the two institutions.

### Aims of the Study

Decision-making and enacting change in medical education are strengthened by evaluative processes [14]. This study aimed to evaluate the utility of the program as perceived by trainees using both quantitative and qualitative measures. A secondary aim was to explore fellows’ perceptions of pediatric cardiology training and its challenges.

## Methods

Individuals were eligible to participate in the study if they were in fellowship or higher specialist training in pediatric cardiology in either institution and had attended at least one session. Trainees in Ireland are referred to as specialist registrars, but the term fellow is used interchangeably throughout the text for consistency. A semi-structured questionnaire was administered online using the SurveyMonkey platform. Trainees were sent a link to their email inviting them to participate. All data, including protected health information, were anonymized, and personal information was not collected. Data were stored in accordance with GDPR guidelines.

**Fig. 2:**
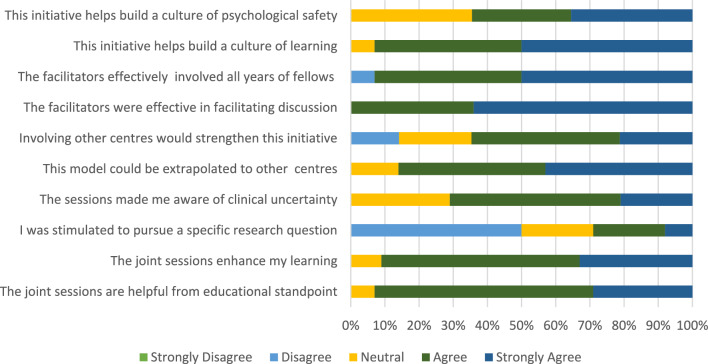
Responses to questions graded on Likert scale

Closed questions are listed in Fig. 2 and Tables 2 and 3. Open questions are listed in the supplementary material. The questions related to demographic information, utility of joint sessions, practice variation, and clinical uncertainty, challenges and limitations of the program, and educational experiences of fellows. Both closed and open questions were utilized. Closed question format included Likert scales; ranked preferences; and multiple-choice questions. Quantitative statistical analysis was performed on responses to closed questions, and responses were expressed as mean and standard deviation or percentage of response in case of Likert scales. Ranked items were aggregated in order to compile an ordered list. Statistical analysis was performed using Microsoft Excel.

**Table 2:** Closed questions related to the joint educational sessions

Which sessions were most helpful? (please rank)
- Early VSD Closure vs PA Band
- Yasui vs Norwood in Aortic Atresia with Ventricular Septal Defect and balanced ventricles
- Endocarditis and percutaneous pulmonary valve implantation
- Decision-making in management of common congenital heart lesions
- Blalock–Thomas–Taussig shunt vs right ventricular outflow tract stenting in Tetralogy of Fallot with hypercyanotic spells
- History of congenital heart disease
- Cardiac transplantation challenges: PTLD and antibody mediated rejection
- Pulmonary atresia intact septum with coronary sinusoids: management dilemmas
- Antenatal management of hypoplastic left heart syndrome
- Other
What was the best part of each session? (please rank)
- The discussion
- Highlighting areas of uncertainty
- The lecture
- Ability to ask questions

**Table 3:** Fellows experience as learners

How do you learn best? (please rank)
Clinical practice
Lectures
Books
MDT conference
Journal club
Journals
Workplace assessments
If in your final year do you feel ready to transition to faculty practice?
Strongly agree 33%
Agree 33%
Neutral 33%
Disagree 0%
Strongly disagree 0%
How often do you undergo assessment of your training?
6 monthly: 75%
Annually: 25%
What type of assessment do you undergo?
OSCE
Bedside examination
Workplace assessments (e.g., Mini CEX)
MCQs
Entrustable Professional Activities
Other
Do you receive the following feedback on your assessments?
Oral: 50%
Written: 83% Any: 83%

### Qualitative Analysis

The following qualitative terms are defined for readers who may be unfamiliar with their use.

- Grounded theory: a qualitative methodology, which begins with no fixed theory. Instead, theories and concepts arise iteratively from the data collected through analysis [15].
- Coding: the process of labeling qualitative data in order to develop themes [16]. Inductive coding refers to a process in which researchers create codes through reviewing the data, in contrast to deductive coding in which a pre-defined list of codes is used. Descriptive coding uses single-term coding when reviewing the data. Consensus coding is performed by two (or more) researchers who review the data individually and compare results to reach a consensus.
- Member checking: a process in which a selection of participants are presented with the research findings and are interviewed for feedback.
- Saturation: a method of ensuring quality in data sampling. Saturation is reached when no new data are generated from analysis of additional sources [17].
- Triangulation: refers to the use of multiple sources of data, allowing the subject to be viewed from multiple viewpoints.

A qualitative analysis was conducted on the open-ended questions using the following steps. Grounded theory methodology was selected as it is well suited to the study of social and educational processes. Theory is developed through analysis of the responses of a group of participants, when their experiences are highlighted to them by the researcher [18]. Responses were coded using inductive, consensus and descriptive style coding conducted by two of the authors until saturation was reached. Central themes were then derived from the initial codes. The following methods of ensuring validity were employed. Descriptive validity was achieved through triangulation. Data source triangulation was achieved through comparison of responses from Dublin and Texas [19]. Interpretative validity was established using verbatim quotations and through member checking. Member checking was conducted as per McKim et al. Four participants (2 from each center) were presented with a draft of the findings followed by structured questioning [20] with all finding their thoughts and experiences well captured. Further use of verbatim quotations was requested by members in the final manuscript.

This study conforms with the principles outlined in the Declaration of Helsinki. Ethical approval was granted by The Research and Ethics Committee of Children’s Health Ireland at Crumlin and Texas Children’s Hospital.

## Results

### Demographic Information

In total, 6 participants from CHI and 20 from TCH were invited to participate. Fourteen fellows (54%) participated in the study. Ten fellows (71%) were from Texas Children’s Hospital, all of whom were originally from the USA, while four were from Children’s Health Ireland (29%) all of whom were originally from Ireland. The first 4 years of fellowship were evenly distributed with 4 fellows in year 1 and 3 in years 2–4; there was one fellow in year 5. Median number of sessions attended was 5 (interquartile range 3–6).

### Utility of Joint Sessions

**Fig. 3:**
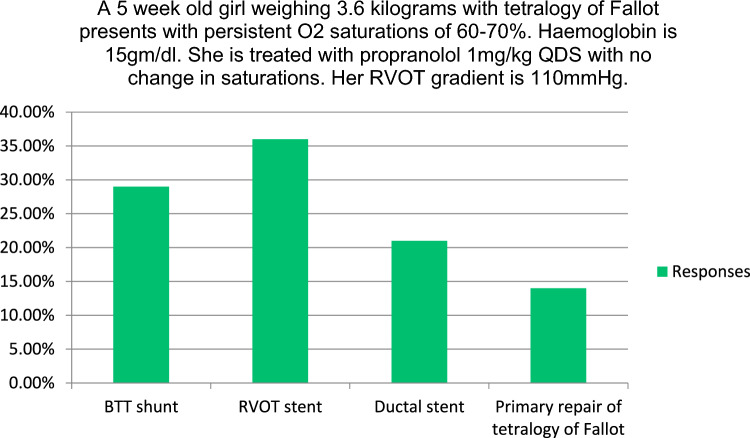
Management of the infant with Tetralogy of Fallot and significant cyanosis. *Ductal stent if ductus remains open

**Fig. 4:**
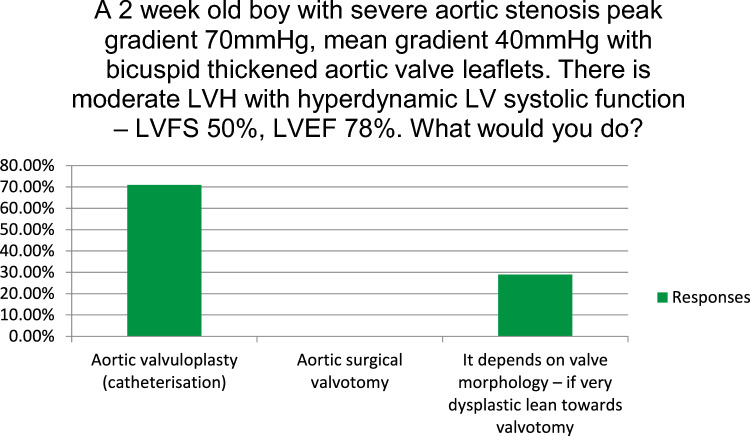
Management of neonatal aortic stenosis

The closed questions related to the joint educational sessions and their responses are listed in Fig. 2 and Table 2. The majority (93%) found the sessions helpful from an educational standpoint, with the remainder neutral (7%). Respondents were asked to rank 10 session topics in order of their utility. The topics are listed in Table 2. The highest ranked session was a discussion of anonymized clinical cases of ventricular septal defects which differed in their management between the USA and Ireland, while the second highest ranked session dealt with the management of aortic atresia with VSD and normal biventricular dimensions. Both aimed to generate constructive debate and highlighted complex management decisions with elements of clinical uncertainty. Respondents were asked to rank what they found to be the most useful component of the sessions (Table 2) and the discussion was ranked highest. The majority (71%) of respondents agreed that the sessions highlighted areas of clinical uncertainty in practice. Fellows were asked multiple choice questions on management of clinical case vignettes. Their responses are depicted in Figs. 3 and 4. Notable is the variation in practice among the group on the optimal approach to patient management.

### Challenges and Areas for Improvement

All responses are listed in Table 2. Fifty percent of respondents found it difficult finding time to attend sessions. The majority (81%) cited clinical commitments as the main barrier to attendance, with conflicting schedules. An aim of the sessions was to stimulate fellows to develop a research question (either between institutions or individually). Twenty-nine percent agreed that the sessions had inspired a research question, while 50% disagreed. Respondents provided free-text commentary on suggested changes and improvements to the session. More frequent sessions, utilization of technology-enhanced learning solutions, and fellow-led sessions were suggested. Eighty-six percent agreed or strongly agreed that this model of learning could be extrapolated to other international centers. Sixty-five percent of respondents agreed that involving other centers (for example, from South America, Asia, or Australia) would strengthen their experience of the program as a learner.

### Fellows as Learners

The respondents were asked closed questions regarding their experience as learners throughout their training with responses listed in Table 3. When asked to rank learning resources by utility the highest ranking was clinical practice itself. The lowest ranking was work-based assessments. Final-year fellows were asked if they felt ready to transition to faculty practice. 66% percent agreed or strongly agreed, while 33% were neutral.

### Qualitative Analysis

Following two rounds of inductive, consensus and descriptive style coding conducted by two authors, saturation was reached after 12 interviews. 34 individual codes were identified. From the 34 codes, 3 central themes were derived which were practice variation; managing uncertainty; and cognitive overload.

#### Practice Variation

The concept that the educational experience of the fellows was enhanced by the contrasting practices of the other institution emerged as a clear theme from within the responses. Respondents were asked to provide commentary on the practice in the other institution. Participants noted the variation in practice between the two centers. Specifically highlighted was a preference for interim and definitive catheter-based interventions over surgical techniques in Dublin when compared with Texas. Several trainees cited right ventricular outflow tract stenting compared with BTT shunt in the management of Tetralogy of Fallot. Fellows noted a preference for earlier surgical repair of ventricular septal defects in Texas when compared with Dublin. Also noted was the institutional preference in Texas for infants with high-risk lesions such as hypoplastic left heart syndrome to remain in hospital between stages 1 and 2. Fellows from both institutions emphasized that they had never considered the management practices of the other institution. There was a sense of mutual respect in the commentary. Neither expressed criticism of the other’s practice but rather saw it as an alternative means of providing excellent care.

“Centers may practice things differently for different reasons but ultimately have similar results”

Cultural differences (for example, patient’s choice of their physician in Texas) and resources (comparatively fewer in Dublin) were suggested as sources of practice variation.

“It was nice to see another center, which is bigger than ours, doing things similarly and validating for me personally that I am achieving a good standard of training for my future career.”

#### Cognitive Overload

The theme of cognitive overload was expressed by learners in discussing the challenges facing during their fellowship.

“….trying to absorb as much as I can but knowing I’m at the tip of the iceberg”

Time management (in balancing arduous clinical commitments with research, scholarship, and home life) was frequently referenced. Learners additionally perceived the breadth of material required to be a significant obstacle.

“Trying to better grasp some of the more granular details that factor into decision making, my default is generally to defer to the opinions of my supervisors or those with more experience.”

#### Managing Uncertainty

The theme of uncertainty, and how to manage it, was common across several lines of questioning. Firstly, regarding the challenges fellows faced in training, cited was the complexities of working in a field with limited randomized control trial evidence.

“It was challenging how often we came back to the lack of good evidence regarding many of our clinical decisions in pediatric cardiology.”

This was a source of clinical uncertainty. Participants also referenced uncertainty in their career and their path to becoming a consultant/attending. Individual codes within this theme were the sense of imposter syndrome and concerns around job security. Promisingly, free-text commentary from final years when compared with other participants referenced an embrace of the uncertain;

“Not there yet, but I think I will feel that ‛one never feels ready’ and that having the modesty to accept that even once finished training you are always learning.”

### Concept Map

**Fig. 5:**
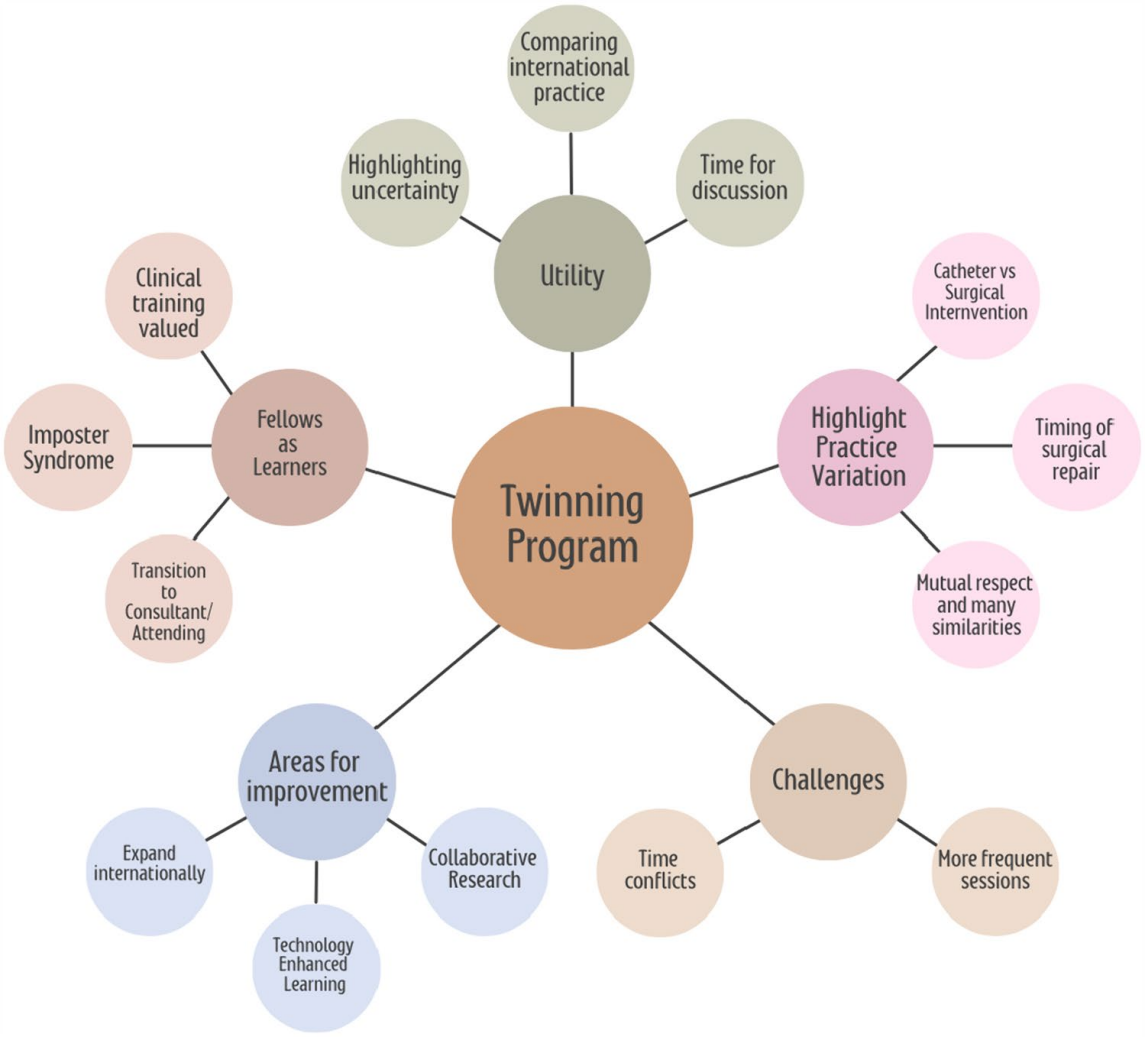
Conceptual map of international pediatric cardiology fellowship twinning

A concept map (Fig. 5) was created to encapsulate the key findings of the questionnaire.

## Discussion

To our knowledge, this is the first description of a sustained educational twinning partnership between international pediatric cardiology training programs described in the literature. Beyond the core competencies of a trainee, there is progressive interest in developing self-regulated, adaptive learners who can think critically and manage clinically uncertain situations [10, 21, 22]. One of the central premises of the program is the tacit acknowledgment that pediatric cardiology is a burgeoning medical specialty in which randomized control trial data are limited and clinical uncertainty is common [21, 23]. Exposure to another program in a different country with different resources and ways of tackling problems may expand trainee horizons on how to care for patients and their families. Through feedback and coaching, the sessions aimed to instill in learners critical reasoning and awareness of clinical uncertainty [4]. Feedback on the utility of the sessions from learners was overwhelmingly positive. Fellows valued the case-based format, with ample time for discussion both with their peers and with facilitators.

“This session is one of the best learning experiences so far in my fellowship.”

The concept that exposure to uncertainty is beneficial within medical education is not novel. Indeed, inclusion of such experiences in curricula has been recommended as a means of introducing threshold concepts within education. Threshold concepts may be considered novel ways of viewing a problem which allow learners to alter their perception of a discipline leading to progression and mastery [24]. The qualitative analysis highlighted fellows felt the sessions exposed them to uncertain situations encouraging critical thinking.

Published literature on the perspectives of pediatric cardiology trainees themselves regarding their training is limited. One published survey from Germany highlighted a lack of clarity regarding expected competency outcomes, achievability of quota-oriented curricula and incorporation of research (largely performed outside of working hours) as challenges. Regular feedback and educational sessions were deemed essential [25]. An explorative qualitative assessment of first year American fellows’ fears while starting their training highlighted increased responsibility as the most common concern compounded by a steep learning curve, clinical uncertainty, and burnout [26]. These findings are broadly echoed in this qualitative exploration, with trainees citing time management and balance as the biggest challenges to their training. The central theme was one of cognitive overload which highlighted the competing demands on time with the needs of a huge specialty conceptually. Interestingly, trainees ranked more traditional learning methods (namely clinical practice and lectures) above work-placed assessments in terms of their educational utility. There have been recent calls to re-assess established competency-based milestone assessments with an entrustable professional activity (EPA) framework within assessment of pediatric cardiology. EPAs are a tool in which learners are assessed on their ability to independently carry out a task. They are therefore task oriented, operational, and centered in clinical practice itself as opposed to conceptual competency domains [27]. EPAs may align more closely with trainee expectations.

### Areas for Future Development

One of the aims of the program is to encourage and enhance connections between the two institutions is to foster collaborative research. The program alignment with this aim was evaluated here. Twenty-nine percent of trainees reported that the sessions had inspired a research question which held promise. Encouraging collaborative research between international institutions involves commitment and investment. Conducting international research in the field of pediatric cardiology has long been a challenge and randomized control data are lacking [28]. However, evidence from the multi-center Single-Ventricle Reconstruction Trial, for example, shows us just how transformative collaborative research can be [28, 29]. The authors hope that collaboration between these two centers will encourage international research in future.

The program utilized existing technologies resulting in a model that is readily adaptable to institutions internationally. The majority (86%) of respondents agreed that this framework could be expanded internationally, with approximately two-thirds considering that the program would be strengthened by an additional international partner, including from a low- or middle-income country. eLearning technologies and online modules have been embraced within pediatric cardiology [30–33] in expanding educational opportunities internationally. For example, Heart University aims to be a source of reliable online resource for e-Learning within CHD [30]. As described by Ma and Vervoort, leveraging e-Learning solutions in low- or middle-income countries requires careful consideration of operability and compatibility [31].

### Limitations

The study is limited by small sample size and participation rate of 56%. Of note, with regard to nationality, all fellows were from either the USA or Ireland. The experience of international fellowship trainees would strengthen the responses. Questionnaires as a chosen method of data collection have several potential pitfalls. Misunderstanding or nuance within the answers may be missed. Furthermore, both the questions and answers are required to be brief. More rigorous but time-consuming qualitative methods such as semi-structured interviews may have yielded richer responses from participants given limited space for free text commentary and pre-determined questions.

## Conclusion

This two-center international twinning partnership demonstrates an effective online educational collaboration between two pediatric cardiology centers in the USA and Europe. The potential for deep learning was highlighted through the use of educational sessions that centered around case-based presentations followed by discussion of practice variation and agreement between the institutions, which were chaired by experienced pediatric cardiologists from both centers. Trainees overwhelmingly found the sessions to be of educational utility particularly as they provided time for discussion and highlighted areas of clinical uncertainty. Areas for future development include embracing technology enhanced learning, encouraging collaborative research, and international expansion.

## Supplementary Information

Below is the link to the electronic supplementary material.

Supplementary file1 (DOCX 14 KB)
