# Supplementary material for: Twinning International Pediatric Cardiology Fellowship Programs: A Transformative Educational Experience for Trainees with Potential for Global Adoption
Source: Pediatr Cardiol. 2024 Apr 2;46(3):580–9. doi: 10.1007/s00246-024-03469-x (PMC11842398; doi:10.1007/s00246-024-03469-x)
Supplement: Supplementary file 1 — Supplementary file1 (DOCX 14 KB) [file 246_2024_3469_MOESM1_ESM.docx]

| **Open-ended questions** |
| --- |
| What practice in the other centre opened your eyes to alternative ways of treating patients? |
| Did any areas of practice preference/bias emerge from the other centre (e.g. RVOT stent compared to BTT shunt, type of Fontan procedure, difference in pregnancy management for HLHS etc.) |
| Which areas of uncertainty did you become aware of? |
| What challenges do you find most significant at your stage of training? |
| Do you have any concerns regarding future practice as a cardiologist attending/consultant? |
| What additional educational strategies would help in your education/training? |
| What changes to the fellowship twin sessions do you think would help? |
| Were you aware of the other centre (Houston/Dublin) prior to participating in the joint sessions? |
| What surprised you about the other centre (Houston/Dublin)? |
| What more could the facilitators do to improve this experience? |
